# Supplementary material for: Fetal Aortic Blood Flow Velocity and Power Doppler Profiles in the First Trimester: A Comprehensive Study Using High-Definition Flow Imaging
Source: Bioengineering (Basel). 2024 Apr 15;11(4):378. doi: 10.3390/bioengineering11040378 (PMC11048424; doi:10.3390/bioengineering11040378)
Supplement: Supplementary file 1 [file bioengineering-11-00378-s001.zip › bioengineering-2901850-supplementary.pdf]

**Supplement**

**Table S1:**

**Pearson Correlations of birth weight (BW) and the Aortic PS, ED, Ns, ISI, IFI, NT, CRL, and MA in 24 live births.**

| | BW | ISI | Ns | PS | ED | PS+ED | IFI | MA | NT | CRL | GP (%) |
|---|---|---|---|---|---|---|---|---|---|---|---|
| BW | 1 | 0.023 | 0.114 | 0.118 | -0.025 | 0.098 | -0.110 | 0.383 | 0.431* | 0.395 | -0.251 |
| ISI | | 1 | 0.677** | -0.508* | -0.327 | -0.508* | -0.035 | -0.363 | 0.222 | -0.229 | -0.413* |
| Ns | | | 1 | 0.242 | 0.168 | 0.245 | -0.096 | -0.283 | 0.337 | -0.001 | -0.137 |
| PS | | | | 1 | 0.575** | 0.986** | -0.147 | 0.179 | 0.147 | 0.364 | 0.381 |
| ED | | | | | 1 | 0.704** | 0.713** | -0.031 | 0.200 | 0.317 | 0.359 |
| PS+ED | | | | | | 1 | 0.019 | 0.149 | 0.168 | 0.381 | 0.405* |
| IFI | | | | | | | 1 | -0.135 | 0.109 | 0.100 | 0.134 |
| MA | | | | | | | | 1 | 0.090 | 0.177 | 0.065 |
| NT | | | | | | | | | 1 | 0.353 | 0.230 |
| CRL | | | | | | | | | | 1 | 0.183 |
| GP (%) | | | | | | | | | | | 1 |

*. Correlation is significant at the 0.05 level (two-tailed). GP (%): Growth Percentile

**. Correlation is significant at the 0.01 level (two-tailed).
